# A Biomaterial-Based Approach to Trypsin Sensing: Design and Optimization of Gelatin–Casein Films

**DOI:** 10.1021/acsomega.5c03938

**Published:** 2025-08-12

**Authors:** Chinaza Ogbonna, Youngjin Kwon, Ka Ram Kim, Woon-Hong Yeo, Nima Ghalichechian, Luke Beardslee

**Affiliations:** † George W. Woodruff School of Mechanical Engineering, 115724 Georgia Institute of Technology, Atlanta, Georgia 30332, United States; ‡ Wearable Intelligent Systems and Healthcare Center (WISH Center), Institute for Matter and Systems, Georgia Institute of Technology, Atlanta, Georgia 30332, United States; § School of Electrical and Computer Engineering, 1372 Georgia Institute of Technology, Atlanta, Georgia 30332, United States; ∥ Institute for Matter and Systems, 1372 Georgia Institute of Technology, Atlanta, Georgia 30332, United States

## Abstract

Real-time postoperative monitoring systems have tremendous potential to detect postoperative complications faster before patients become systemically ill. This study investigates the potential of gelatin–casein blend films as a biodegradable, implantable biomaterial platform for trypsin detection, which is a potential biomarker for an anastomotic leak from the duodenum or proximal jejunum. Although implantability has not been verified in this case, the implantability of gelatin and casein-based biomaterials is substantiated by their demonstrated cytocompatibility as evidenced below and established utility in medical applications, as evidenced by recent advancements in biomaterials research. We systematically evaluated nine gelatin–casein blends, ranging from 0:100 to 100:0 ratios to optimize their enzymatic degradability and response, establishing a material platform for future biosensing applications. Optimal refers to the sensor’s ability to rapidly detect physiological concentrations of trypsin in the body while generating the maximum detectable response. Characterization of the films was performed using Fourier-Transform Infrared Spectroscopy (FTIR), UV–visible Spectroscopy (UV–vis), and Quartz Crystal Microbalance (QCM). The films’ responses to trypsin were analyzed through limit of detection, initial reaction rates, and absorbance shifts. For statistical analysis, a flexible exponential decay model, was employed to assess the significance of the results. Our findings reveal that the 75:25 casein:gelatin blend exhibits superior performance, with the lowest limit of detection (7.81 × 10^–11^ M), highest initial reaction rate (6.936 ΔHz/s by QCM, −0.095 AU/min by UV–vis), and most significant absorbance shift (−2.208 AU after 10 min). This optimal blend demonstrates a 10-fold improvement in detection limit compared to pure gelatin films and a 5-fold enhancement over pure casein films. The remarkable sensitivity, rapid response, and significant signal change of the 75:25 casein:gelatin blend make it a promising candidate biomaterial platform for an implantable trypsin sensor.

## Graphical abstract

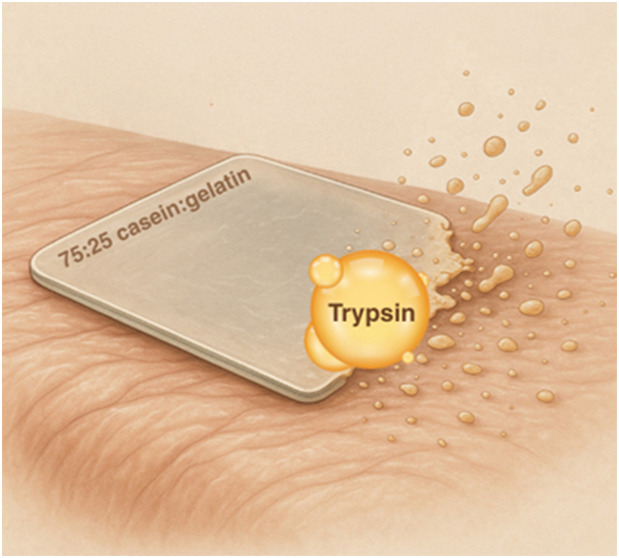

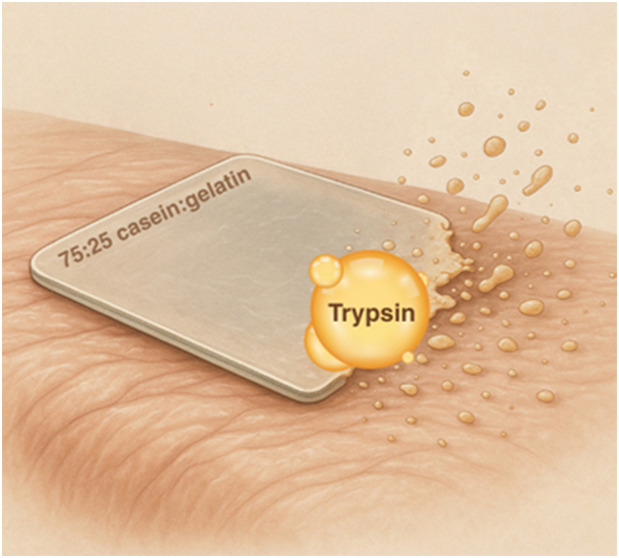

## 1 Introduction

Anastomotic leakage is an unfortunately all too common complication following gastrointestinal surgery with ileocolonic leakages occurring in up to 3% of operations.^1^ These new connections are commonly made during a variety of surgeries including gastric bypass, resection of tumors in the proximal small intestine, during surgeries for ulcers, and after trauma among others. This failure of the surgical connection can lead to peritonitis, sepsis, and potentially death, with mortality rates ranging from 1.5% to 12%.^1^ Early detection of anastomotic leakage is crucial for timely intervention and improved patient outcomes.

Detection of leaks from the gastric-jejunal or jejunal–jejunal anastomosis in gastric bypass surgery involves a combination of clinical assessment, imaging studies, and laboratory tests. Clinically, signs such as fever, tachycardia, abdominal pain, and nausea are critical indicators, with leaks often presenting within an average of 2 days postsurgery.^2^ The delay in the appearance of symptoms following a leak often results in reactive diagnostics and treatment, leading to worse patient outcomes. Imaging techniques, particularly computed tomography (CT) scans with oral contrast, are considered the most effective, boasting detection rates as high as 86%.^3^ While laboratory tests such as an elevated white blood cell count can suggest a postoperative issue, they are inherently nonspecific and can be due to a variety of factors.^4^ Ultimately, no single method guarantees 100% efficacy; thus, a comprehensive approach combining clinical suspicion and various diagnostic tools is essential for early and accurate identification of leaks. Lack of early detection of anastomotic leakage increases the clinical and economic burden on both patients and hospitals.^1–4^

Trypsin, a serine protease secreted by the pancreas into the duodenum, plays a key role in protein digestion. Its presence in the abdominal cavity can serve as an indicator of anastomotic leakage, particularly in upper gastrointestinal surgeries. It is important to note that under normal physiological conditions there should be no pancreatic trypsin present in the abdominal cavity. While various methods exist for trypsin detection, including colorimetric,^5^ fluorometric^6,7^ and mass spectrometry-based techniques,^8^ their application in anastomotic leakage detection faces challenges such as sensitivity and their ability to be incorporated into an implantable format, which can be read out remotely. While current enzyme-responsive materials like DNA-functionalized nanoclusters, nanoporous membranes, and synthetic polymer films show promise for trypsin detection, they face limitations such as poor biocompatibility, lack of biodegradability, and high manufacturing costs. This study leverages natural protein blends to address these gaps.

Recent research has explored the use of protein-based films, particularly those made from gelatin^9–12^ and casein,^13–17^ as potential sensing materials for various applications. These natural polymers offer advantages such as biodegradability, biocompatibility, and the ability to form films with tunable properties.^15^

Trypsin interacts differently with the triple-helix structure of gelatin (a derivative of collagen) and the micellar structure of casein. Gelatin’s resistance to trypsin^18^ is primarily due to its intact triple-helix, though mutations or structural disruptions can increase enzymatic sensitivity. In contrast, casein micelles^19^ undergo a more complex degradation process, characterized by bond hydrolysis, micelle rearrangement, and secondary structure changes. These structural differences influence the degradation kinetics and performance of gelatin–casein blends, which can be optimized for applications requiring sensitive enzymatic response. Figure 1 illustrates the response of casein and gelatin to trypsin. Trypsin’s effects on gelatin are often localized, with site-specific sensitivity influenced by structural mutations, such as those in fibronectin-binding domains.^20^ These properties suggest that gelatin targets enzymatic response to the blend, ensuring controlled degradation.

**Figure 1:**
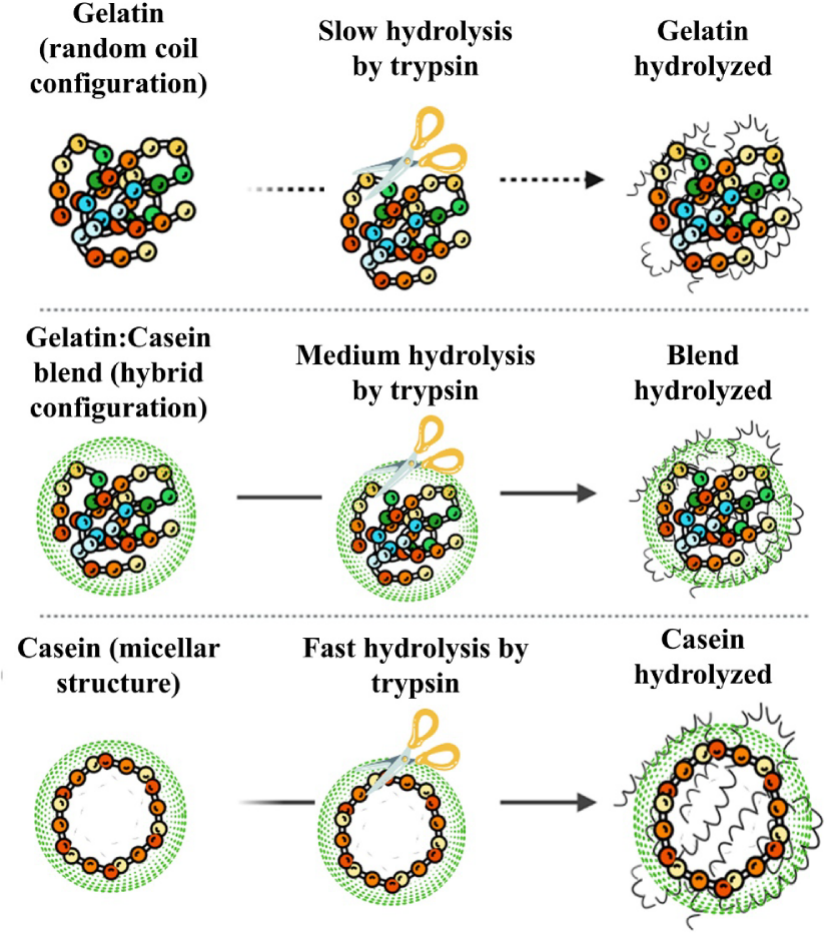
Schematic illustration highlighting the interaction between the protein blend and trypsin.

Casein micelles, in contrast, are highly dynamic under trypsin activity. Proteolysis induces hydrolysis of β-casein bonds, micelle rearrangement, and the formation of nanoparticles or low-molecular-weight peptides.^19^ The proteolysis process progresses through distinct kinetic stages, altering the micellar organization and secondary structure. This dynamic degradation enhances the sensitivity of casein-dominated blends to enzymatic activity.

By combining these proteins, an optimal blend will demonstrate ideal enzymatic degradability and secondary structure reorganization. Casein contributes porosity and elongation properties, while gelatin enhances structural integrity and water resistance. The resulting films exhibit increased porosity from casein’s emulsifying properties, leading to air incorporation and a porous matrix,^21^ a balance of flexibility and elongation, with moderate tensile strength,^15^ and enhanced sensitivity and faster enzymatic response due to accessible cleavage sites and hybrid secondary structures. The primary objective of this work is to optimize casein:gelatin blends for use in implantable enzymatic sensing platforms, with a focus on functional performance—namely enzymatic detection sensitivity, degradation kinetics, and film behavior in aqueous environments.

Enzyme-responsive protein-based films offer promising potential for developing innovative biomaterial platforms capable of detecting enteric content leakage into the abdominal cavity. The creation of such enzyme-responsive materials represents a crucial advancement in biosensing applications. While our investigation specifically demonstrates trypsin detection, the potential responsiveness of these films to other proteases present in duodenal fluid could enhance their reliability in detecting anastomotic leakage.

Through systematic evaluation of these films, we aim to elucidate their enzymatic degradability, secondary structure, and mechanical properties—characteristics critical for future sensor integration. This research proposes a biodegradable, implantable material capable of real-time trypsin detection, effectively bridging the gap between conventional diagnostic approaches and the pressing need for rapid, localized, and minimally invasive detection of postoperative leaks. By harnessing enzymatic degradation to produce a measurable response, these films address a critical unmet need in early, point-of-care diagnostics for anastomotic leakage.

While this work is foundational, the findings lay the groundwork for the future development of biosensing systems that rely on the degradation-dependent response of these materials. Subsequent efforts will focus on integrating these films into sensor designs, validating their performance in clinical scenarios, and assessing their behavior under realistic physiological conditions.

## 2 Materials and Methods

### 2.1 Chemicals

Materials used in this study include technical-grade casein from bovine milk (CAS 9000-71-9) and gelatin from porcine skin (CAS 9000-70-8), both sourced from Sigma-Aldrich (St. Louis, MO, USA). Trypsin from porcine pancreas (CAS 9002-07-7) and Nα-Benzoyl-l-arginine 4-nitroanilide hydrochloride (BAPNA; CAS 21653-40-7) were also obtained from Sigma-Aldrich. Additional reagents included Tris buffer (1 M, MW: 121.14), glycerol, cellulose triacetate, and glutaraldehyde, all purchased from Millipore Sigma (Burlington, MA, USA). Quartz Crystal Microbalance (QCM) sensors with a gold coating (Q-Sense, QSX 301, ∼4.95 MHz fundamental frequency) were sourced from Biolin Scientific (Gothenburg, Sweden).

### 2.2 Sample Preparation

Trypsin solutions (0.001–1000 ng/mL) were prepared by dissolving trypsin in a 1:1 mixture of Tris buffer (pH 8.0) and phosphate-buffered saline (pH 7.4) at room temperature, followed by purification through a 0.2 μm syringe filter. A 5% (w/v) gelatin solution was produced by dissolving gelatin in deionized water at 50 °C and cooling to room temperature. Casein was dissolved in distilled water at room temperature to achieve a 10% (w/v) solution, adjusted to pH 7.0 with sodium hydroxide, and mixed with 30% (w/v) glycerol plasticizer at 80 °C for 10 min. The solution was then heated to 85 °C for 15 min in a temperature-controlled water bath and cooled to room temperature.

Casein–gelatin blends were synthesized at various ratios (87.5:12.5, 75:25, 62.5:37.5, 50:50, 37.5:62.5, 25:75, 12.5:87.5) by mixing appropriate volumes of the formulated casein and gelatin solutions.

Films were created by depositing 40 μL of either gelatin, casein, or blend solution onto QCM sensor surfaces and spin-coating at 5000 rpm for 30 s to achieve a thickness of approximately 250 nm. Film thickness was measured using a Tencor P15 Profilometer (Keyence, Osaka, Japan). Films were dried under ambient conditions and set overnight at 4 °C.

The necessity of cellulose triacetate (2% w/v) for adhesion and glutaraldehyde (0.5%) for cross-linking was evaluated but deemed unnecessary for this study as the goal is to obtain reliably rapid film degradation. The degradation of gelatin is reduced by glutaraldehyde cross-linking and cellulose triacetate blending, though through distinct mechanisms. Glutaraldehyde chemically stabilizes gelatin through covalent cross-links,^22^ while cellulose triacetate acts as a physical barrier to enzymatic and hydrolytic degradation.^23^ Figure S1 illustrates the frequency response normalized to the pH 5-to-trypsin transition (1,000 μg/mL) comparing gelatin, gelatin cross-linked with glutaraldehyde, and gelatin cross-linked with glutaraldehyde and adhered using cellulose triacetate. The curve shows a faster and more significant degradation with gelatin alone than with the addition of glutaraldehyde and cellulose triacetate. Furthermore, the goal of this study is to identify the optimal blend—defined as a composition that maximizes the sensor’s ability to rapidly detect physiological trypsin concentrations while producing the highest measurable response—excessive degradation resistance from glutaraldehyde and cellulose triacetate is not desirable. Additionally, our films adhered well enough to the sensor surface.

### 2.3 Fourier Transform Infrared Spectroscopy (FTIR)

Chemical interactions between casein and gelatin blends were assessed using a spectrometer (Thermo Scientific Nicolet iS50 FTIR, Waltham, Massachusetts, USA). Spectra were collected using 32 scans per sample with a spectral resolution of 4 cm^–1^ and a data spacing of 0.482 cm^–1^ at room temperature. Samples were incubated in a water bath for 10 min before 150 μL was deposited on the sample compartment for analysis. The final spectra were recorded in % transmittance format. Prior to each measurement, background spectra were collected using 8 scans. The spectral acquisition range was 650–4000 cm^–1^. The instrument was equipped with a deuterated triglycine sulfate (DTGS) detector and a potassium bromide (KBr) beamsplitter. An infrared (IR) source and a Smart iTR accessory with a diamond crystal window were employed for attenuated total reflectance (ATR) measurements. Additional instrumental parameters included a gain setting of 1, an optical velocity of 0.4747, and an aperture of 80.

### 2.4 UV–Vis Spectrophotometry

Trypsin activity was quantitatively analyzed using UV–vis spectrophotometry (Thermo Scientific Evolution 220, Waltham, Massachusetts, USA) by measuring absorbance from 350 to 800 at 1 nm intervals before and after trypsin addition (0–60 min) using a quartz cuvette with a bandwidth of 1 nm, integration time of 0.1 s, data interval of 1 nm, scan speed of 600 nm/min and each run took an estimated time of 51.6 s.

Nα-Benzoyl-l-arginine 4-nitroanilide hydrochloride (BAPNA) is a commonly used chromogenic substrate for measuring trypsin activity. When trypsin cleaves BAPNA, it releases *p*-nitroaniline, which has a yellow color and absorbs light at 405 nm.^24^ The native absorption peak of proteins, including trypsin, is typically around 280 nm due to aromatic amino acids. By using BAPNA as a substrate, the assay shifts from measuring trypsin directly (at 280 nm) to measuring the product of trypsin activity (*p*-nitroaniline at 405 nm).^25^ To mitigate light scattering effects caused by casein-induced turbidity while still capturing *p*-nitroaniline formation, 449 nm wavelength was chosen. Reaction mixtures contained 150 μL BAPNA, 1000 μL of protein solution (gelatin, casein, or casein:gelatin blends at ratios of 87.5:12.5, 75:25, 62.5:37.5, 50:50, 37.5:62.5, 25:75, 12.5:87.5), and 600 μL of 1000 ng/mL trypsin solution. Samples were incubated at 37 °C. Initial reaction rates were determined from linear regression of the first 10 min of trypsin activity.

### 2.5 Quartz Crystal Microbalance (QCM) Analysis

A QCM (Biolin Scientific Q-Sense E4) was used to determine the degradation kinetics of trypsin with various substrates. Degradation experiments were performed using a pH 5 buffer solution as a baseline, flowing through the cell for 5 min prior to introducing trypsin solutions. All experiments were conducted at 37 °C with a flow rate of 0.1 mL/min.

### 2.6 Data Analysis

A flexible exponential decay model similar to this^26^ was employed, allowing the fitting of nonlinear decay patterns with adaptable parameters. This model emerged as the most effective approach due to its balance between flexibility and simplicity, its ability to fit the data accurately, and its provision of interpretable parameters (e.g., decay rates and *p*-values).

### 2.7 Film Preparation

Films composed of a 25:75 gelatin–casein blend (75C25G) were prepared by depositing 0.5 mL of the blend solution onto an aluminum substrate and drying in a vacuum oven (Heraeus Vacuum Oven) at 50 °C for 1.5 h. Following fabrication, the films were stored at 4 °C. To assess stability over time, one batch of films was analyzed immediately after preparation, while a second batch was stored for 28 days prior to characterization.

### 2.8 Mechanical Testing

The tensile strength and elongation tests were conducted using a Mark-10 force gauge (Model: Series 5, Mark-10 Corporation, Copiague, NY, USA) equipped with a motorized test stand (Model: ESM303). The system was calibrated with a load cell of 50 lbf capacity (equivalent to 222.4 N), and the tests were performed at a controlled pulling speed of 1 mm/min. Rectangular specimens with dimensions of 0.5 cm in length and 1.2 cm in width were used, with a gauge length of 20 mm.

### 2.9 Cytotoxicity Testing Chemicals and Apparatus

To assess cytocompatibility, metabolic activity was evaluated using the MTS assay, which measures mitochondrial enzyme activity as an indicator of cell viability and proliferation. This assay reflects overall cellular function rather than direct cell adhesion to the material surface. A bioresorbable 5 w%v polycaprolactone (PCL) coating was applied to the blends to modulate degradation rates and maintain structural integrity during testing. NCTC clone 929 (L929) cell and Eagle’s minimum essential medium (EMEM) was purchased from ATCC (VA, USA). Fetal bovine serum (FBS), and penicillin–streptomycin (PS) was purchased from Gibco (NY, USA). The 3-(4,5-dimethylthiazol-2-yl)-5-(3-carboxymethoxy-phenyl)-2-(4-sulfophenyl)-2H-tetrazolium (MTS) reagent, a cytotoxicity test solution was purchased from Promega (WI, USA). A sodium phosphate-buffered saline (PBS) solution (50 mM) containing 0.15 M NaCl (PBS, pH 7.2) was purchased from Corning (NY, USA). Humidified CO_2_ incubator, water bath, centrifuge, and biosafety cabinet were utilized during the cell culture process. Bio-Tek plate reader instruments (WA, USA) were applied to read the absorbance from MTS reaction.

*Mus musculus*’s subcutaneous connective tissue cell L-929, was chosen to be assessed as a cytotoxicity test. Specific complete medium for cell culture includes EMEM (ATCC, USA) supplemented with 10% fetal bovine serum (Gibco, USA) and 1% penicillin–streptomycin (Gibco, USA). L929 cells are cultured in a humidified 5% CO_2_ atm incubator at 37 °C.

### 2.10 TGA/DSC Analysis

The thermal stability of the synthesized polymers was examined by thermogravimetric analysis (TGA). The test method for TGA was as per ASTM E-1131-20, using SDT-Q600 (Simultaneous DTA-TGA, TA Instruments Inc., USA). Alumina cups were used as sample and reference containers and calcined alumina was used as the reference material. The samples were analyzed from room temperature to 60 °C under nitrogen purging at a heating rate of 5 °C/min, then 60 to 300 °C under nitrogen purging at a heating rate of 10 °C/min.

## 3 Results and Discussion

### 3.1 FTIR

While FTIR offers detailed information on changes in the secondary structure, UV–vis and QCM are more sensitive to molecular-level changes affecting absorbance and mass, respectively. We examine the hydrolysis of casein–gelatin blends by trypsin after 10 min of incubation, highlighting the differences in sensitivity among these techniques and their implications for interpreting hydrolytic processes.

Figure 2 illustrates the FTIR spectroscopic analysis of casein–gelatin blends before and after trypsin treatment which reveals significant structural modifications across multiple spectral regions. This comprehensive analysis focuses on the Amide A (3200–3300 cm^–1^), Amide I (1600–1700 cm^–1^), Amide II (1500–1600 cm^–1^), and the Amide III bands (C–N stretching, N–H bending: 1230–1240 cm^–1^; β sheet structures: 1260–1280 cm^–1^; random coil conformations: 1300–1330 cm^–1^), providing insights into changes in hydrogen bonding, protein secondary structure, and local molecular environments. Figure S2 exhibits the FTIR spectra of all blend solutions before trypsin was added, 10 min after incubation with 1,000 μg/mL trypsin at 37 °C, as well as the comparison of the 75C25G optimal blend before and after 10 min of trypsin exposure.

**Figure 2:**
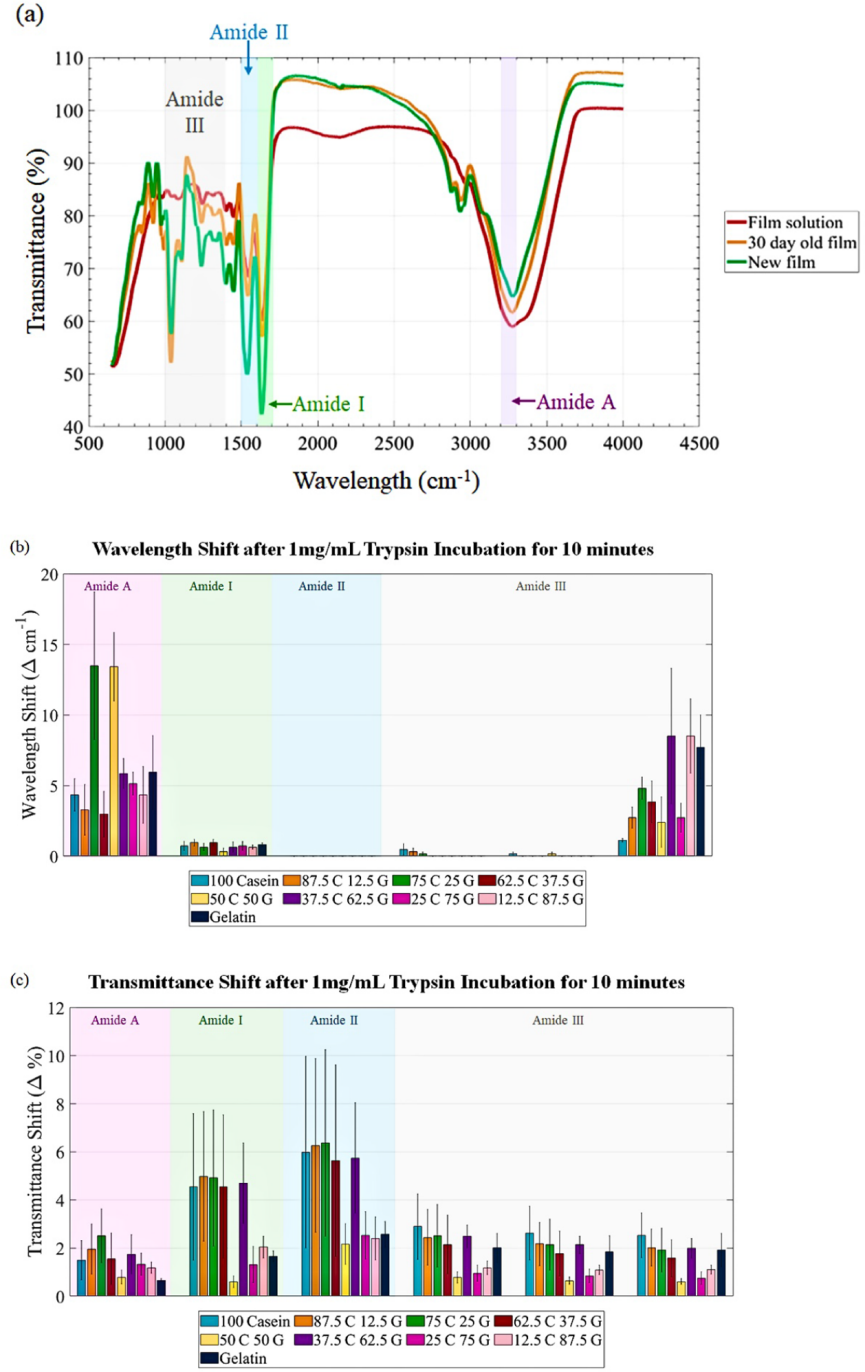
FTIR spectra: (a) Comparison of the spectra for the film solution, 28-day-old, and 0-day-old 75C25G films. (b) Wavelength shifts across all amide regions for all protein blends. (c) Transmittance shifts across all amide regions for all protein blends. Error bars are the standard error $\left(\mathrm{SE}=\mathrm{STD}/\sqrt{\#\mathrm{samples}}\right)$ . The shifts are indicated by (*X*_end_ – *X*_start_).

#### 3.1.1 Amide A Region (3200–3300 cm^–1^)

The Amide A band, associated with N–H stretching vibrations,^27^ exhibited significant shifts across most blends, the 75:25 casein:gelatin blend (75C25G) shows the largest wavelength and transmittance shifts in this band, shifting from 3287.13 cm^– 1^ to 3273.63 cm^–1^ (Δ13.50 ± 5.23 cm^–1^), and at the peak wavelength for transmittance, 3222.039 cm^–1^, shifted by 2.51 ± 1.12%. The larger shift in the 75C25G blend suggests more extensive alterations in hydrogen bonding networks, likely due to its optimal composition for trypsin activity.

#### 3.1.2 Amide I Region (1600–1700 cm^–1^)

The Amide I band, crucial for secondary structure determination,^28^ showed significant shifts and new peak formations, with the 87.5C12.5G blend demonstrating the most substantial change closely followed by the 75C25G blend. The 87.5C12.5G blend exhibits the largest transmittance shifts in this region with the wavelength shifts being insignificant as it is less than the resolution of the assessment, at the peak wavelength for transmittance, 1640.67 cm^–1^, shifted by 4.97 ± 2.69%. However, the 75c25g was a close second with a transmittance shift of 4.92 ± 2.82% and then 100% casein with a transmittance shift of 4.55 ± 3.05%. These are all within 2% of each other. This is consistent with casein’s known susceptibility to hydrolysis by trypsin, which primarily affects the backbone and secondary structure. Casein’s open, disordered structure without the stabilizing effect of gelatin allows trypsin to readily disrupt the C=O stretching and N–H bending, resulting in pronounced shifts in this region. The pronounced shift in the casein dominant blends indicates a more dramatic transition from α-helical to β-sheet or disordered structures,^29^ highlighting its unique responsiveness to trypsin hydrolysis.

#### 3.1.3 Amide II Region (1500–1600 cm^–1^)

The Amide II band, reflecting N–H bending and C–N stretching vibrations,^27^ displayed both shifts in existing peaks and the emergence of new peaks, with the 75C25G blend showing the most comprehensive changes The 75C25G has the highest transmittance shift (Δ 6.38 ± 3.87% at 1539.91 cm^–1^) as well as new peak at 1543 cm^–1^, suggesting that this composition most effectively destabilizes the backbone in response to trypsin, leading to a noticeable change in transmittance. The absence of wavelength shifts in the Amide II band across all blends could indicate that the N–H bending and C–N stretching vibrations associated with this band are less responsive to changes in secondary structure induced by trypsin. In FTIR, wavelength shifts generally reflect alterations in the energy levels of specific molecular bonds or structures, often resulting from substantial changes in the protein’s hydrogen bonding or overall conformation.

The Amide II band is often less sensitive to secondary structure changes compared to Amide I and Amide III, as it primarily reflects in-plane bending of N–H groups rather than stretching vibrations.^30,31^ Since casein and gelatin have inherently flexible, disordered structures,^32,33^ the addition of trypsin may not induce a structural rearrangement significant enough to shift the vibrational frequency within this band. Instead, the response is primarily detected through transmittance shifts, which reflect intensity changes but not frequency shifts.

Moreover, the lack of wavelength shifts in the Amide II band across all blends suggests that trypsin’s action primarily affects bonds that contribute more directly to backbone cleavage and secondary structure reorganization (as observed in Amide I and Amide III bands) rather than the bending motions captured in Amide II. This aligns with trypsin’s specificity for peptide bonds,^33^ which may impact the hydrogen bonding network around the Amide I and III bands more noticeably than the Amide II band.

Thus, the stable wavelength in the Amide II band across all blends may reflect the inherent uniform activity of the N–H bending vibrations in response to enzymatic action, with minimal alteration to their energy states compared to other regions.

This significant shift in the existing peak and the emergence of a new peak indicates more extensive structural reorganization compared to other blends.

#### 3.1.4 Amide III Region (C–N Stretching, N–H Bending: 1230–1240 cm^–1^; β Sheet Structures: 1260–1280 cm^–1^; Random Coil Conformations: 1300–1330 cm^–1^)

This region, associated with CH_2_ bending, C–N stretching, and Amide III vibrations,^27^ revealed significant changes. Interestingly, the 37.5C62.5G blend has the largest wavelength shift in the Amide III random coil region (8.52 ± 4.80 cm^–1^) and is closely followed by 12.5C87.5G (8.52 ± 2.62 cm^–1^), and then gelatin (7.71 ± 2.27 cm^–1^) which reflects the unique impact of gelatin. Gelatin, being partially denatured collagen, retains random coil characteristics that respond more significantly in terms of wavelength shifts when interacting with trypsin. However, for transmittance shifts in the Amide III random coil band, 100% casein shows the highest shift, possibly due to the complete disruption of any remaining ordered structures in casein under trypsin activity, which gelatin’s more stable structure partially resists.

### 3.2 Comparative Analysis and Significance

In terms of differential sensitivity across spectral regions, Amide A and Amide III regions showed significant wavelength and transmittance shifts, indicating changes in hydrogen bonding and secondary structure. Amide I and II regions displayed transmittance shifts but no significant wavelength shifts, suggesting less sensitivity to secondary structure changes. Amide III region revealed complex changes, with different blends showing maximum shifts in different subregions.

For blend-specific significance, the 75C25G blend showed the most comprehensive changes across multiple regions (largest shifts in Amide A and II, significant shifts in Amide I and III), suggesting an optimal composition for trypsin activity. Casein, being a more disordered protein, likely contributes to increased accessibility for trypsin.^34^ Gelatin, with its partially renatured collagen-like structure,^35^ may provide additional cleavage sites or alter the overall blend structure in a way that enhances trypsin activity.

Overall, structurally, the shift in Amide A indicates transitions from β-sheet to α-helical disordered backbone conformations. This dramatic shift (tens of cm^–1^) in Amide A usually reflects a major conformational rearrangement or a qualitative change in bonding.

The uniform activity of Amide II wavelengths across all blends suggests that N–H bending and C–N stretching vibrations are less affected by trypsin-induced structural changes. Changes in the Amide III region provide information on alterations in CH_2_ bending, C–N stretching, and local conformations. The extensive shifts in the Amide A and III regions in the 75C25G blend provide compelling evidence for more comprehensive transitions from β-sheet to α-helical disordered structures upon trypsin hydrolysis.

The comprehensive FTIR analysis reveals that trypsin hydrolysis induces significant structural changes in casein–gelatin blends, with the 75C25G blend exhibiting the most pronounced and diverse spectral shifts. Our findings contribute to a nuanced understanding of protein blend hydrolysis dynamics and have important implications for the design of enzyme-responsive biomaterials with tailored degradation profiles, particularly highlighting the potential of the 75C25G blend for applications requiring heightened sensitivity to enzymatic activity.

### 3.3 UV–Vis Spectrophotometry

The analysis of various casein–gelatin blends revealed a complex interplay between protein composition and enzymatic activity. The ratio of casein to gelatin significantly influenced the response to trypsin, with each blend exhibiting unique characteristics:

Casein-dominant blends (≥75% casein) showed rapid initial changes followed by quick stabilization. This behavior can be attributed to the micellar structure of casein, which provides numerous accessible cleavage sites for trypsin. Casein micelles are not solid spheres, but rather have a porous, sponge-like structure which allows enzymes like trypsin to penetrate the micelle to some extent, accessing more than just the surface proteins.^34^ Casein micelles are in a constant state of dynamic equilibrium, with individual casein molecules and small aggregates continually associating and dissociating. This dynamic nature means that even casein molecules initially in the core can become exposed to the surface and thus to enzymatic attack. κ-casein, which forms the outer layer of the micelle, is particularly susceptible to enzymatic cleavage which can destabilize the micelle, making the interior more accessible.^36^
Gelatin-dominant blends (≥62.5% gelatin) exhibited more prolonged and sometimes cumulative effects. This may be due to the triple-helix structure of gelatin, which unfolds gradually under enzymatic attack, exposing new cleavage sites over time.
Balanced ratios (37.5–62.5% of each protein) demonstrated nonlinear responses, suggesting complex interactions between the proteins and the enzyme. This could be due to the formation of a hybrid network structure, where casein micelles are interspersed within the gelatin matrix.

Figure 3 and Table 1 exhibit the emergence of the 75C25G blend as the optimal composition, exhibiting the largest absorbance shift after 10 min of trypsin exposure. This blend likely provides an ideal balance of accessible cleavage sites and uniform enzymatic activity, enhancing trypsin detection sensitivity. Figure S3 demonstrates the absorbance spectra of 75C25G from pretrypsin addition to 10 min postaddition, collected at 1 min intervals and the spectral comparison of all blends over time within 400 nm to 500 nm. When compared to pure casein or gelatin substrates, the blends generally showed enhanced performance for trypsin detection:

Pure casein exhibited rapid initial degradation but quick stabilization, limiting its sensitivity over extended periods.
Pure gelatin showed minimal changes after trypsin addition, with consistent but slight changes over time, indicating lower sensitivity.
The optimal 75:25 casein:gelatin blend outperformed both pure proteins, demonstrating a 13% larger absorbance shift than the next best blend.

**Figure 3:**
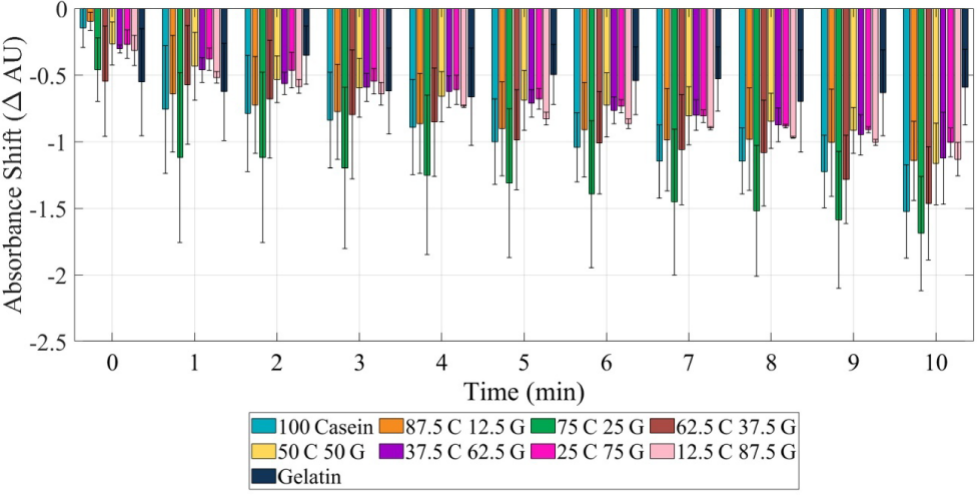
Absorbance shift over first 10 min after trypsin is added to the protein blend 75:25 casein:gelatin blend has the most significant shift across all 10 min.

**Table 1:** Figures of Merit for Protein Blends Developed in This Work^a^

**Gelatin Content**	**Casein Content**	**Limit of detection** (ng/mL)	**Initial reaction rate** (ΔHz/s)^a^	**Absorbance shift** (10 min)	**Decay rate** (AU/min)	***p***-Value^a^
0	100	21.31	6.87	–0.87	21.52	0.01
12.5	87.5	24.14	2.6	–1.14	21.04	0
25	75	1.82	6.94	–1.69	17.92	0
37.5	62.5	19.6	2.34	–1.46	1.76	0
50	50	59.18	3.53	–1.17	0.71	0
62.5	37.5	4.4	1.74	–1.12	0.33	0
75	25	71.03	1.65	–1.01	0.27	0.01
87.5	12.5	2.1	1.32	–1.13	0.02	0
100	0	3.39	0.14	–0.59	0.02	0

a The initial reaction rates, expressed in AU/min, were measured via UV–vis spectrophotometry, while the reaction rates in ΔHz/s were determined using QCM.

These results suggest that the combination of casein and gelatin creates a synergistic effect, enhancing the substrate’s overall sensitivity to trypsin activity.

### 3.4 Kinetics of Trypsin Activity

The trypsin-induced behavior of the blends followed a characteristic pattern:

All blends exhibited an immediate response upon the addition of trypsin. The most significant changes occurred within the first 2 min, particularly in the casein-dominant blends. Trypsin activity peaked at various times depending on the composition of the blend: blends with 75% or more casein reached peak activity within 1 min, blends with 37.5–62.5% casein peaked between 3 and 5 min, and those with 12.5% or less casein peaked around 10 min. After reaching peak activity, the systems began to gradually stabilize, likely due to factors such as substrate depletion, product inhibition, or alterations in the properties of the blend that affected enzyme accessibility. The nonlinear changes observed over time suggest complex reaction kinetics, potentially involving multiple phases or substrates.

Tables S1 and S2 show the statistical analysis (ANOVA) was conducted to substantiate the small variations in UV–vis absorbance. This ensures that any observed differences exceed the expected instrumental noise or baseline drift. Analysis of Variance (ANOVA) was used to determine the significance of the response over time compared to the absence of trypsin.

The flexible exponential decay model similar to this^26^ was applied to analyze the temporal response dynamics of nine different samples across ten time points (Time 0 to Time 10) following the addition of trypsin. The primary objective was to identify the decay rates and most critical time points for each sample after Time 0.

The flexible exponential decay function, which accommodates multiphase decay patterns, was selected for its capacity to better represent complex degradation kinetics compared to simpler exponential formulations. The fitting process involved minimizing the residual sum of squares between the observed data and the predicted values through iterative optimization. This approach allows for a more nuanced description of the degradation dynamics, capturing both rapid initial changes and subsequent slower decay phases that are often observed in protein-based materials.

The analysis focuses on two key parameters: decay rates and *p*-values. Decay rates, measured in AU/min, indicate how quickly each sample’s response diminishes over time. A higher decay rate suggests faster degradation when exposed to trypsin. *P*-values represent the statistical significance of the changes observed at each time point after the initial measurement (Time 0). They are calculated by comparing the actual measured values against those predicted by our decay function. In interpreting the results, samples with higher decay rates, such as 75C25G blends with a rate of 17.92 AU/min, show a rapid decrease in response following the addition of trypsin, indicating quick degradation. Conversely, samples with lower decay rates, like 100% gelatin with a rate of 0.023 AU/min, exhibit a more gradual change in response, suggesting slower degradation or different reaction kinetics.

The flexible exponential decay model successfully captured the varied decay behaviors across different samples, revealing distinct degradation kinetics and critical time points for each sample. The results from this study can be found in Table 1.

Our findings underscore the importance of using flexible models for analyzing complex biochemical processes, where kinetics may involve multiple phases or nonlinear dynamics. Future work may explore further model refinements or alternative approaches to more precisely characterize the underlying mechanisms of these responses.

### 3.5 QCM

Trypsin activity evaluation was conducted through degradation experiments following a standardized protocol. A pH 5 buffer solution, nonreactive to both casein and gelatin, was utilized as a baseline, flowing through the cell for 5 min prior to the introduction of trypsin solutions. All experiments were performed at 37 °C with a constant flow rate of 0.1 mL/min. The film degradation rate was quantified by analyzing the slope of the linear portion of the frequency change versus time curves as illustrated in Figure 4a, which varied as a function of enzyme activity. Figure 4b exhibits the degradation rates of the diverse blends to varying trypsin concentrations. The experiments were performed at the fundamental frequency, with *n* = 3 indicating three independent replicates to ensure measurement reliability and reproducibility.

**Figure 4:**
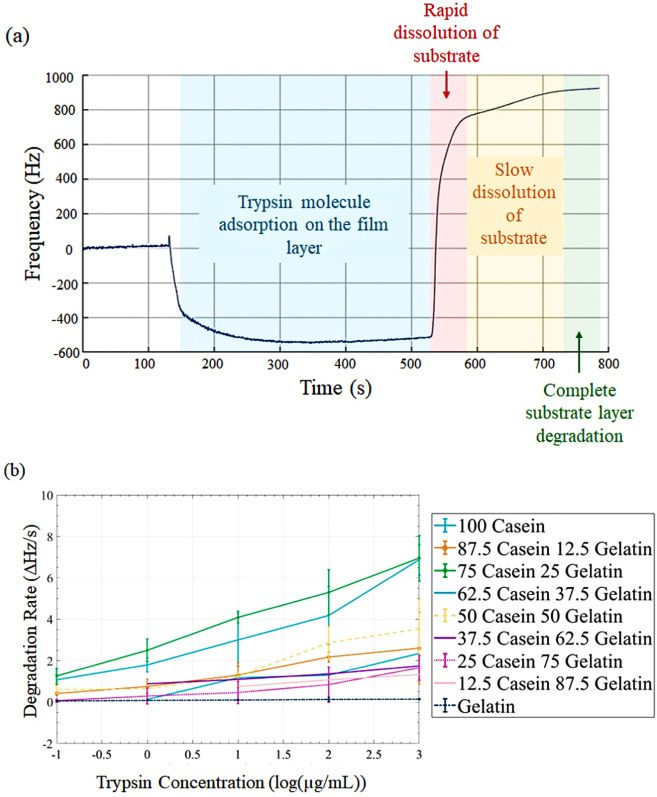
QCM (*n* = 3): (a) frequency–time response of the 75C25G blend under degradation by 1000 μg/mL trypsin. (b) Degradation rate curves for six protein blends across a range of trypsin concentrations from 0.001 μg/mL to 1000 μg/mL.

The initial frequency decrease, indicative of mass increase, was attributed to enzyme molecule adsorption on the film layer, initiation of enzymatic reaction, and additional film swelling. Subsequent frequency increase signaled the dissolution of degradation products. The additional film swelling observed can be attributed to two primary mechanisms: increased hydration and structural modifications. As enzymatic degradation progresses, cleavage of polymer chains exposes hydrophilic functional groups, enhancing the film’s affinity for water. This leads to increased water uptake, contributing to film expansion. Furthermore, enzymatic activity disrupts the polymer’s internal network, generating void spaces within the film. These voids can be occupied by water molecules, further contributing to swelling.^37^ Complete substrate layer degradation was evidenced by the stabilization of the QCM frequency. This degradation model effectively interpreted the observed phenomena, particularly evident in the QCM response to low-activity trypsin.^11^ In this case, film layer degradation was delayed for over 5 min postenzyme exposure, especially in films with higher gelatin content. Conversely, at higher enzyme activities, the frequency decrease was negligible due to rapid film degradation. The degradation rates shown in Figure 4b are based on the initial rapid response to trypsin. This is why casein has the highest response as explained from the UV–vis results. Casein had a rapid initial response followed by a very quick stabilization. The average initial reaction rate over the first 10 min after trypsin exposure was also determined for a one-to-one comparison with the UV–vis results as illustrated in Table 1. The system was not washed with a pH 5 buffer at the end of the experiment, as the film had fully degraded from the QCM sensor surface by that point. However, we acknowledge that the transition from pH 5 to pH 8 may introduce bulk effects due to changes in viscosity and ionic strength, potentially influencing the sensor’s frequency response. To assess this, a comparison of the 75C25G blend, 5 w/v% polycaprolactone, and a blank sensor at 1 μg/mL trypsin is included in the Supporting Information (Figure S4). The data show no significant frequency change during the transition from pH 5 to the trypsin solution in the control scenarios.

Since proteins degrade gradually in buffered solutions without trypsin, direct controls on degradation rates remain challenging. However, if residual buffer conditions influence degradation kinetics, this should be considered when interpreting the results.

The decision to report reaction rates in ΔHz/s instead of mass/s is due to the inability to determine certain film properties such as density. The Sauerbrey equation is unsuitable for accurately determining the thickness of protein films due to its underlying assumptions, which require the film to be thin, rigid, and uniformly distributed. While the Voigt model is more appropriate for viscoelastic films, its application depends on the precise determination of the film’s density, which is challenging for these spin-coated thin films. A calibration curve for trypsin activity evaluation was constructed over a range of 0.001 μg/mL to 1000 μg/mL (4.3 × 10^–5^ M to 4.3 × 10^–11^ M), as presented in Figure 4b. The limits of detection (LOD) were determined for various casein:gelatin blends and shown in Table 1 by dividing three times the standard deviation of the blank by the slope of the calibration curve. To confirm the optimal blend, the LOD for 87.5:12.5 casein:gelatin was also determined, yielding 7.81 × 10^–11^ M (1.82 ng/mL). These results corroborated that the 75C25G blend exhibited the lowest LOD, thus confirming it as the optimal composition for trypsin detection. This blend successfully completely covers the clinically relevant range for trypsin levels in, for example, normal patients (11 ± 4 nM),^6^ and partially cover that for chronic renal failure (47 ± 25 nM)^5^ and chronic pancreatitis conditions (60 ± 27 nM)^6^ and is comparable to other sensors found in literature (Table 2).

**Table 2:** Comparison of the Optimum Blend in This Work to Similar Trypsin Detection Systems Reported in the Literature^a^)

	**Response time (min)**	**Limit of detection** (ng/mL)	**Sensing mechanism**	**Implantability**
This work (optimum blend)	Real-Time	1.82	Quartz Crystal Microbalance	Yes
Reference 6		1.4	Nanopore	No
Reference 7		1	Fluorescence	No
Reference 36		60	Internal Reflection Spectroscopy	No
Reference 37		4.5	Fluorescence	No
Reference 38	Real-Time	58.25	Electrochemical	No

a Reference 6 utilizes a biological alpha-hemolysin protein nanopore,reference 7 employs DNA-stabilized silver nanoclusters, reference 36 features urease and fluorescein5(6)-isothiocyanate (FLITC) immobilized on nanoporous anodic alumina (NAA), reference 37 integrates protamine with amino-modified silicon nanoparticles (SiNPs) and GSH-capped goldnanoclusters (GSH-AuNCs), and reference 38 uses casein and BSA.

This study characterizes the structural and functional properties of gelatin–casein blend films, emphasizing their enzymatic responsiveness to trypsin. The distinct degradation behaviors of gelatin and casein highlight their complementary roles in the blend.

The triple-helix structure of gelatin provides inherent resistance to trypsin, which serves as an indicator of structural integrity. However, local disruptions, such as glycine substitutions,^20^ increase sensitivity by destabilizing hydrogen bonding networks.

The enhanced proteolytic degradation of gelatin–casein blended films by trypsin arises from distinct structural and biochemical interactions between the two proteins. Compared to single-protein films, the blend exhibits increased enzyme accessibility, facilitated by matrix porosity, complementary substrate specificity, and mutual stabilization of enzymatic activity.

The disruption of gelatin’s triple-helix domains, coupled with the inherently disordered conformation of casein, facilitates deeper enzyme diffusion. Pure gelatin films, in contrast, resist enzymatic hydrolysis due to their tightly packed collagen-like structure, necessitating prior denaturation for effective protease action. Casein incorporation mitigates this limitation by introducing structural disorder, thereby improving trypsin accessibility.

In addition to structural modifications, the gelatin–casein blend promotes enzymatic synergy by offering complementary cleavage sites. Trypsin preferentially hydrolyzes lysine- and arginine-rich regions within gelatin, while its activity toward casein is largely directed at serine- and threonine-containing motifs. Within the blended matrix, casein acts as a sacrificial substrate, undergoing rapid hydrolysis and generating localized disruptions that expose gelatin regions to subsequent enzymatic action.^5^ This effect is absent in single-component films, where protease activity is constrained to specific amino acid sequences, limiting degradation efficiency. Prior studies on β-casein-coated surfaces further support this model, demonstrating that casein enhances trypsin-mediated hydrolysis by preventing substrate aggregation and maintaining enzyme accessibility.^38^

As the primary objective of this work is to optimize casein:gelatin blends for use in implantable enzymatic sensing platforms, with a focus on functional performance, our experimental design prioritized evaluating these application-relevant parameters over detailed structural or crystallographic analysis. While XRD and related structural methods would certainly contribute to a more complete understanding of the material properties, such investigations fall outside the current scope but represent a promising avenue for future research. Detailed crystallographic insight will be pursued in followup studies using surface-sensitive methods (e.g., GIWAXS or synchrotron SAXS/WAXS) to relate nanoscale ordering to mechanical and sensing performance.

Beyond facilitating enzymatic cleavage, blended films may promote more consistent enzymatic activity. While our study did not directly measure trypsin stability, QCM and UV–vis experiments suggest that the casein:gelatin matrix enables more uniform enzyme–substrate interaction, which could reduce the localized inhibition seen in single-protein systems. Further studies are needed to confirm whether these blends contribute to long-term enzyme stabilization.

These findings highlight the critical role of gelatin–casein interactions in modulating enzymatic accessibility and degradation behavior. The observed synergy in proteolysis offers new insights into the design of biomaterial systems where controlled enzymatic degradation is desirable, including applications in tissue engineering, drug delivery, and biodegradable scaffolds.

### 3.6 Mechanical Testing

Uniaxial tensile testing was performed on optimized casein–gelatin blend films to evaluate their mechanical properties, comparing freshly prepared films with those aged for 28 days. The tests were conducted at room temperature (approximately 25 °C) under standard laboratory conditions using a Mark-10 Series 5 force gauge and an ESM303 test stand. The force gauge, with a measurement range of 0.01 lbf to 50 lbf and an accuracy of ±0.1% of full scale, provided high-resolution measurements (0.001 lbf or 0.01 N). The test stand allowed precise control of testing speed, ranging from 0.5 mm/min to 500 mm/min, with a load capacity of up to 300 lbf (1,334 N). The elastic modulus (EM) and tensile strength (TS) of the films were calculated using strength-distance data, as the thickness of the films was unknown, preventing stress–strain curve generation.

Table 3 shows that the 28-day-old films exhibited an elastic modulus of 2.01 MPa and a tensile strength of 2.24 MPa, indicating enhanced stiffness and strength with aging. In contrast, freshly prepared films demonstrated an elastic modulus of 1.44 MPa and a tensile strength of 2.13 MPa, reflecting slightly lower mechanical performance. These results suggest a potential increase in cross-linking or structural stability in films with aging. The strength-distance curves provide valuable insights into the mechanical behavior of these biopolymer blends, demonstrating their applicability for intended uses requiring moderate elasticity and strength. In literature, there is limited information specifically addressing the mechanical stability of gelatin or casein films over time. However, the mechanical properties of gelatin^39^ and casein^40^ films are highly dependent on hydration levels. This suggests that changes in environmental humidity over time could impact the mechanical stability of the films.

**Table 3:** Mechanical Properties of the 75C25G Films over Time^a^

	Film-28 day	Film-0 day
Elastic Modulus (MPa)	2.01 ± 0.28	1.44 ± 0.35
Ultimate Strength (MPa)	2.25 ± 0.02	2.13 ± 0.50

a Standard error is expressed by standard deviation across *n* = 3 samples divided by the square root of the number of samples.

### 3.7 Cytotoxicity Measurements

To assess the cytotoxicity of the 75C25G films to L929 fibroblast cells as well as the stability of the cytotoxicity over time, an MTS assay was conducted after 24 h of exposure to the sample extract medium. These films were coated with 5% w/v polycaprolactone to simulate their intended real-world application. The polycaprolactone coating improves mechanical stability and slows the degradation of the sensing film, helping to maintain sensor functionality and ensure reliable signal transmission after implantation. The sample extract medium was prepared by immersing 1.0 × 1.0 cm^2^ film samples in complete cell culture medium at 37 °C for 12 h in an incubator as exemplified by Figure 5a. For the assay, 100 μL of L929 cell suspension at a density of 1.0 × 10^5^ cells/mL was seeded into 96-well plates and incubated for 24 h to allow cell adhesion. Afterward, the culture medium was replaced with sample extract media derived from the 75C25G film samples coated with 5 w%v polycaprolactone. Cells were incubated for an additional 24 h, followed by the addition of 20 μL of MTS reagent to each well. The reaction was allowed to proceed for 1 h, after which the optical density was measured at 490 nm using a plate reader. Each experimental condition was performed in six replicates, and the relative cell viability was calculated as a percentage of the negative control group.

**Figure 5:**
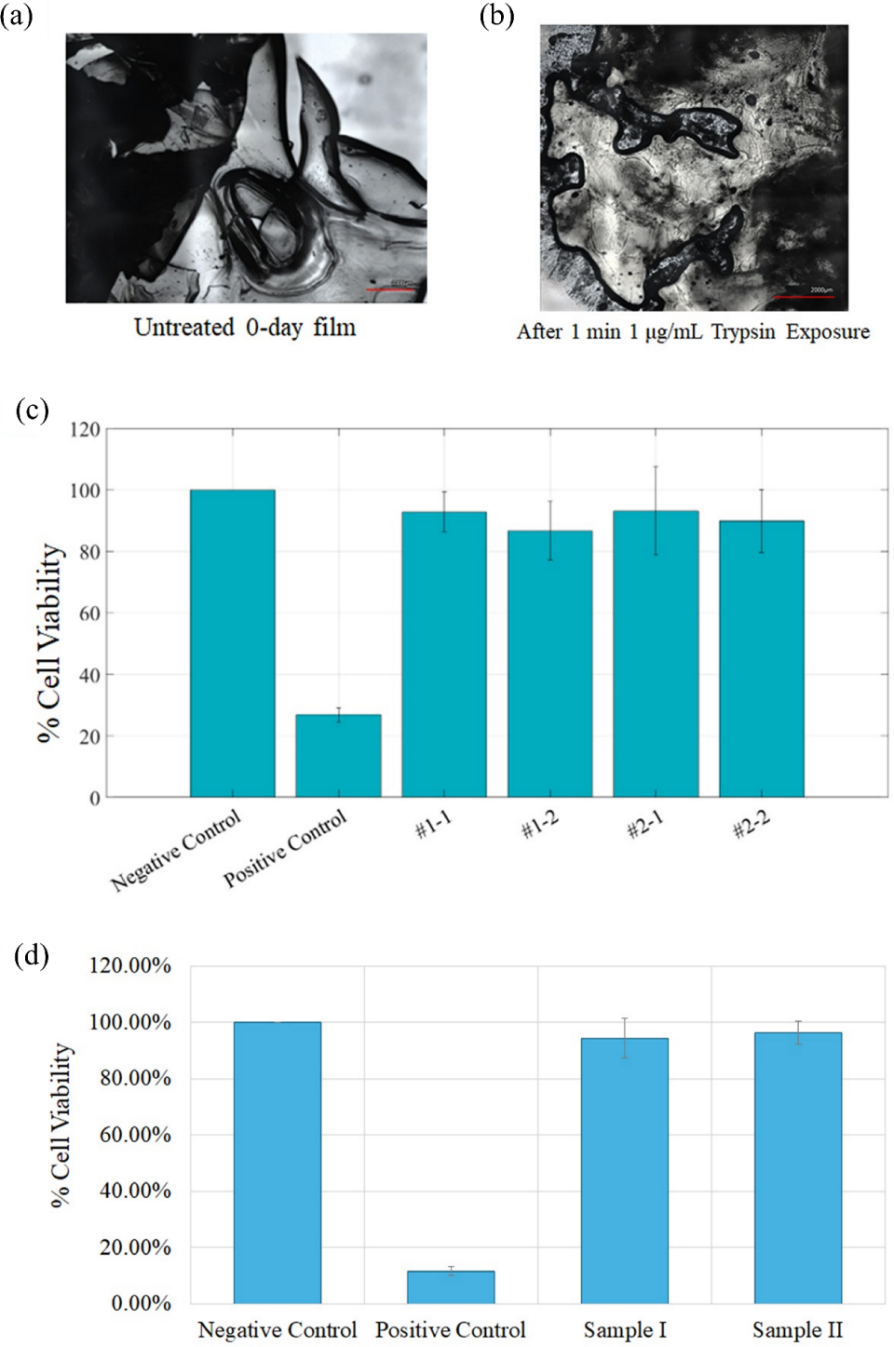
Cytotoxicity measurements. (a) Laser confocal image of the 0-day old 75C25G film untreated. Scale bar = 1000 μm. (b) Laser confocal image after 1 min exposure to 1 μg/mL trypsin solution. Scale bar = 2000 μm. (c) Bar chart comparing cytotoxicity results of the tested samples with negative and positive controls. Sample #1 represents the PCL-coated 28-day-old 75C25G film, while sample #2 corresponds to the PCL-coated 0-day-old 75C25G film. (d) Bar chart comparing cytotoxicity results of the tested samples with negative and positive controls. Sample #1 and #2 represent the 5-day-old 75C25G film.

The negative control group (cells cultured in standard medium without exposure to any materials) exhibited 100% cell viability, confirming the reliability of the assay. In contrast, the positive control (cells exposed to a known cytotoxic agent, 1% SDS) demonstrated significantly reduced cell viability of 26.80 ± 2.24%, validating the sensitivity of the test.

For the 28-day-old films, two replicates yielded average cell viabilities of 92.81 ± 6.61% and 86.66 ± 9.47%. Similarly, the freshly prepared (0-day-old) films demonstrated average viabilities of 93.11 ± 14.27% and 89.88 ± 10.17% across replicates. Both films surpassed the 70% cell viability threshold specified by ISO 10993-5, confirming their noncytotoxic nature.

Slight variability in cell viability was observed across replicates, particularly for the freshly prepared films, which exhibited higher standard deviations. These differences are likely due to minor inconsistencies in sample preparation or localized variations in material properties. Future work will focus on improving film fabrication protocols to enhance uniformity and reduce variability.

Figure 5b shows the purified samples (left), followed by an image capturing the initiation of extraction in complete media, and concluding with the appearance of the films after 24 h of extraction in complete media. As exemplified by Figure 5c, these results highlight the excellent biocompatibility of the 75C25G films modified with polycaprolactone, supporting their suitability for further development as biomaterials.

To assess cytotoxicity of the films without polycaprolactone, the effects of two 5-day old 75C25G films on cellular metabolic activity after 24 h exposure were instigated using the colorimetric MTS assay as recommended in ISO 10993-5. Extracts of each material were prepared at 1 mg mL^–1^ in complete growth medium and allowed to equilibrate for 24 h at 37 °C. A suspension of mammalian cells was adjusted to 5 × 10^5^ cells mL^–1^ and dispensed into 96-well microplates. Immediately thereafter, 100 μL of each extract was added to triplicate wells. Plates were incubated for 24 h under standard culture conditions (37 °C, 5% CO_2_, >95% humidity).

Following exposure, 20 μL of MTS reagent was added to every well and the plates were returned to the incubator for 2 h to allow enzymatic reduction of the tetrazolium salt to its formazan product. Absorbance was measured at 490 nm and normalized to untreated cells grown in parallel, which defined 100% viability. A 0.1% (v/v) sodium dodecyl sulfate solution served as a positive (cytotoxic) control. Images of the exposed samples are in Figure 5e.

Untreated cells exhibited normal metabolic activity and were therefore assigned a viability of 100%. The positive control depressed viability to 11.5 ± 0.008%, confirming the assay could detect severe toxicity and met ISO acceptance criteria. Figure 5f and Table 4 demonstrate the mean normalized viabilities for the test extracts. These were 94.4 ± 0.034% and 96.2 ± 0.021%. Both values exceeded the 70% noncytotoxicity threshold by a wide margin and deviated from the negative control by <6% points, a difference well within typical intra-assay variability for MTS measurements.

**Table 4:** Raw Data of the MTS Assay for the 5-day Old 75C25G Films

**Absorbance**	**1**	**2**	**3**	**4**	**5**	**6**	**Mean**	**SD**
Negative Control	1.781	1.797	1.839	1.889	1.961	2.009	1.879	0.091
Positive Control	0.229	0.203	0.214	0.221	0.217	0.213	0.216	0.009
5-day-old 75C25G film I	1.752	1.755	1.759	1.764	1.775	1.82	1.771	0.025
5-day-old 75C25G film II	1.751	1.767	1.771	1.833	1.842	1.878	1.807	0.051

The high viabilities recorded for both samples indicate that neither material elicited measurable cytotoxicity under the test conditions. The strong response of the Triton X-100 control corroborates the sensitivity of the model system. Because MTS reduction chiefly reflects mitochondrial dehydrogenase activity, the data suggests that the extracts did not compromise cellular energy metabolism or gross membrane integrity during the 24-h exposure.

Several limitations merit consideration. Only a single concentration (1 mg mL^–1^) and exposure period (24 h) were evaluated. A tiered dose–response design extending to lower concentrations and longer incubation times (48–72 h) would provide a more comprehensive toxicological profile and can be considered for future work.

Gelatin and casein-based biomaterials exhibit exceptional cellular compatibility, with in vitro studies confirming favorable fibroblast interactions and negligible cytotoxicity.^41,42^ Their biocompatibility is further validated by in vivo performance in wound healing models, where gelatin/casein hydrogels promoted tissue regeneration without adverse immune responses.^43^

Gelatin’s efficacy in regenerative medicine has been extensively documented, particularly in 3D-printed implants for reconstructive surgery that support cell proliferation and controlled biodegradation.^42^ Casein-based systems show analogous promise, with engineered films demonstrating protease-responsive behavior and radical scavenging capabilities critical for chronic wound management.^41^ Both materials achieve functional versatility through modifiable cross-linking strategies, enabling tunable mechanical properties^41,43^ and degradability profiles aligned with tissue regeneration rates. This evidence base positions gelatin and casein as foundational materials for next-generation implants, though longitudinal in vivo studies remain essential to validate their performance in human physiological environments.

### 3.8 TGA/DSC Analysis

TGA has been used to investigate thermal stability, oxidation, and decomposition reactions of the 75C25G optimum blend. Figure 6 illustrates the TGA curve of 75C25G which shows two weight loss regions, below 180 °C and above 180 °C. The initial weight loss of 18% can be attributed to the loss of moisture and trapped as well as adsorbed water by the gelatin–casein blend. The degradation observed above 180 °C corresponds to the breaking of peptide bonds, leading to the formation of smaller peptide fragments as well as the cleavage of cross-links such as pentosidine and pyridinoline, which are important for the structural integrity of gelatin.^44^ In general, the 28-day film exhibited greater weight loss compared to the 0-day film in the 25 to 60 °C temperature range. This observation suggests that the 28-day storage period may have induced structural changes or facilitated moisture uptake, resulting in increased weight loss upon heating. The 28-day film continued to show more substantial weight loss at higher temperatures compared to the 0-day film. This trend implies that the aging process may have increased the film’s susceptibility to thermal degradation, possibly due to moisture absorption or a reduction in structural stability over time.

**Figure 6:**
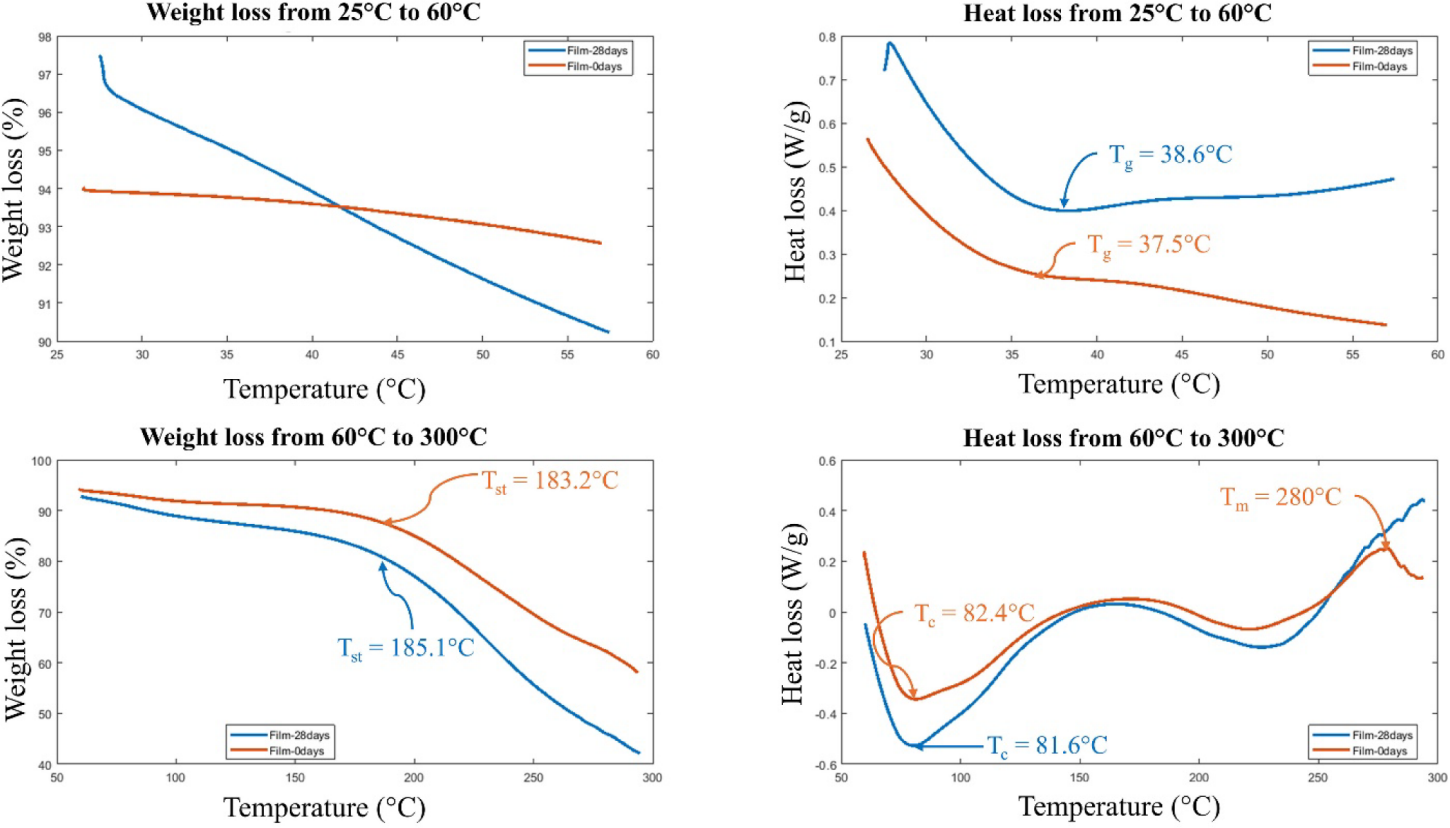
TGA/DSC curves illustrating weight and heat loss from 25 to 300 °C. The blue line represents the 28-day-old 75C25G film, while the orange line represents the 0-day-old 75C25G film. Prolonged storage increases low-temperature stability but renders the blend more prone to thermal degradation at higher temperatures, highlighting moisture-driven structural changes over time.

Figure 6 shows the DSC thermograms of the 28-day old and 0-day old 75C25G films. The thermal scans were conducted in the range of 25 to 300 °C. The glass transition temperature appears around 37–38 °C which seems to be influenced by factors attributed to both gelatin and casein structures. The glass transition temperature of gelatin films depends significantly on water content—for dry gelatin, the *T*_g_ is reported to be around 217 °C^46^ and with increasing water content, the *T*_g_ decreases notably.^46^ In DSC measurements, gelatin films often show two transition temperatures—a lower one attributed to the *T*_g_ of water-plasticized gelatin, and a higher one interpreted as the *T*_g_ of dried gelatin, often superimposed by melting.^45^ Meanwhile, the glass transition temperature of casein is reported to be below room temperature, specifically in the range of 2–30 °C.^48^ This relatively low *T*_g_ is attributed to the destruction of the sample’s structure, which allows for increased molecular mobility and leads to a phase transition.^47^

The DSC curves also show an exothermic crystallization peak at approximately 81–82 °C,^48^ followed by an endothermic melting peak at 280 °C for the 0-day film. For the 28-day film, the melting peak shifts to beyond 300 °C, indicating alterations in thermal behavior induced by storage. Interestingly, the 28-day film demonstrates lower heat loss in the 25 to 60 °C range compared to the 0-day film, suggesting enhanced thermal stability at lower temperatures. However, from 60 to 300 °C, the 28-day film exhibits significantly higher heat loss, particularly at peak transitions, indicative of more substantial thermal events, such as decomposition or structural breakdown, at elevated temperatures.

In summary, the thermal behavior of 75C25G blend films varies significantly with storage time. While the 28-day film shows greater thermal stability at lower temperatures, it undergoes more pronounced decomposition and structural breakdown at higher temperatures, evidenced by increased heat and weight loss. These findings suggest that storage induces changes in moisture content, chemical bonding, and structural rearrangement, resulting in enhanced thermal degradation at elevated temperatures. Understanding these aging effects is critical for optimizing the material’s performance in applications requiring thermal resilience.

By achieving high sensitivity, rapid response, and superior biocompatibility, the gelatin–casein films offer a transformative solution for postoperative monitoring. The material’s implantability allows localized detection of trypsin directly at the surgical site, providing real-time insights that are critical for early intervention and reducing patient morbidity and healthcare costs.

## 4 Conclusion

In conclusion, this study has successfully developed and characterized a series of casein:gelatin blend films, identifying an optimal composition for potential trypsin detection applications. Through systematic evaluation of nine different casein:gelatin ratios, we have determined that the 75:25 casein:gelatin blend offers the most promising composition for future trypsin-responsive materials, demonstrating an optimal balance of sensitivity, mechanical properties, and enzymatic responsiveness.

Our comprehensive characterization using FTIR, QCM, and UV–vis spectroscopy, coupled with robust statistical analysis employing a flexible exponential decay model, provides a solid foundation for understanding the material’s behavior and performance when exposed to trypsin. The 75:25 casein:gelatin blend demonstrated superior performance across multiple parameters, including a remarkably low limit of detection (7.81 × 10^–11^ M), high initial reaction rate (6.936 ΔHz/s by QCM, −0.095 AU/min by UV–vis), and significant absorbance shift (−2.208 AU after 10 min). This optimal blend exhibits a 10-fold improvement in trypsin sensitivity compared to pure gelatin films and a 5-fold enhancement over pure casein films.

The optimized 25:75 gelatin–casein blend not only meets the biochemical and mechanical requirements for enzymatic sensing but also sets the foundation for real-world deployment in clinical settings. Compared to existing enzyme-responsive materials, this blend uniquely combines low detection limits, rapid response, and excellent biocompatibility with implantable and biodegradable properties. These advantages position it as a superior alternative for clinical applications, particularly in resource-constrained settings where manufacturing simplicity and cost are critical considerations.

The insights gained from this study provide critical information for designing degradation-dependent biomaterial platforms, emphasizing their potential for integration into biosensing platforms. Future work will focus on incorporating these films into functional sensors for real-time enzymatic monitoring, evaluating their performance under realistic physiological conditions, and exploring scalability and long-term stability for clinical and industrial use.

By advancing the understanding of these biomaterials, this study paves the way for innovative enzyme-responsive sensing technologies, bridging material characterization with practical biosensing applications. The potential integration of these films into biosensor systems could enable real-time monitoring of critical biomarkers, potentially revolutionizing postoperative care, and patient outcomes.

## Supplementary Material



## Notes

The design and optimization of gelatin–casein films for trypsin sensing; Figure S1: the quartz-crystal microbalance frequency shifts of uncross-linked versus glutaraldehyde-cross-linked gelatin (with or without cellulose-triacetate (CTAC) adhesion) as the medium transitions from pH 5 buffer to 1 mg mL^–1^ trypsin, highlighting the effect of cross-linking and CTAC adhesion on the degradation rates; Figure S2: FTIR spectra of each blend before and 10 min after enzyme exposure, with the 75:25 casein:gelatin (75C25G) formulation displayed in detail to illustrate amide-band changes upon proteolysis; Figure S3: UV–vis absorbance (400–500 nm) for 75C25G at 1 min intervals and compares all blends; Figure S4: 75C25G against polycaprolactone and a blank sensor at trace-level (1 μg mL^–1^) trypsin; Tables S1 and S2: repeated-measures and grand ANOVA results, respectively, confirming significant time-dependent absorbance decreases for most compositions; Table S3: linear regressions that correlate degradation rates with trypsin concentration (*R* ^2^ ≥ 0.94 for optimal blends); together these data substantiate the choice of 75C25G, demonstrate the role of cross-linking, and quantitatively validate the optical and gravimetric responses that underlie the film-based biosensor (PDF)
