# Supplementary material for: A Biomaterial-Based Approach to Trypsin Sensing: Design and Optimization of Gelatin–Casein Films
Source: ACS Omega. 2025 Aug 12;10(33):37597–610. doi: 10.1021/acsomega.5c03938 (PMC12391974; doi:10.1021/acsomega.5c03938)
Supplement: Supplementary file 1 [file ao5c03938_si_001.pdf]

Supplementary Information

A Biomaterial-Based Approach to Trypsin Sensing: Design and Optimization of Gelatin-Casein Films

Chinaza Ogbonna[*1], *Youngjin Kwon*[1,2], *Ka Ram Kim*[1,2], *Woon-Hong Yeo*[1,2], *Nima Ghalichechian*[3], *and Luke Beardslee*[4]

[1] George W. Woodruff School of Mechanical Engineering, Georgia Institute of Technology, Atlanta, GA, 30332 USA

[2] Wearable Intelligent Systems and Healthcare Center (WISH Center), Institute for Matter and Systems, Georgia Institute of Technology, Atlanta, GA, 30332 USA

[3] School of Electrical and Computer Engineering, Georgia Institute of Technology, Atlanta, GA, 30332 USA

[4] Institute for Matter and Systems, Georgia Institute of Technology, Atlanta, GA, 30332 USA

Keywords:

Corresponding Author: Chinaza Ogbonna, chinaza@gatech.edu, (678) 365-1343

Trypsin detection, gelatin, casein, QCM, UV-Visible spectrophotometry, FTiR, cytotoxicity

Author Contributions

The manuscript was written through contributions of all authors. All authors have given approval to the final version of the manuscript.

Supplementary Figures

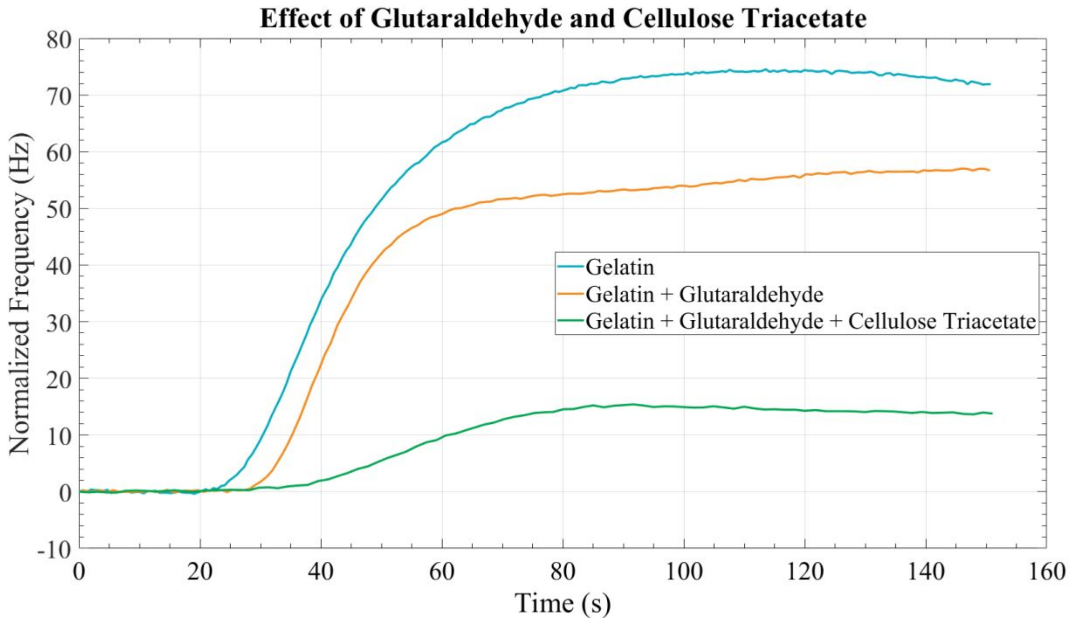

Figure S1. Frequency response normalized to the pH 5-to-trypsin transition (1,000 μg/mL). Comparison of gelatin, gelatin cross-linked with glutaraldehyde, and gelatin cross-linked with glutaraldehyde and adhered using cellulose triacetate.

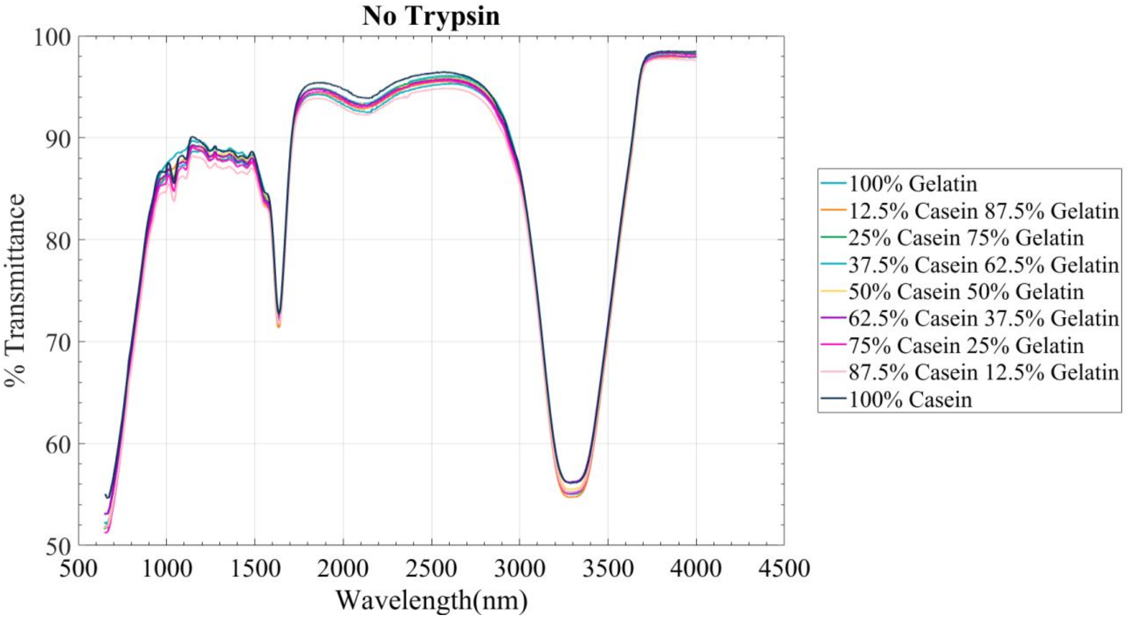

No Trypsin
% Transmittance
Wavelength(nm)
100% Gelatin
12.5% Casein 87.5% Gelatin
25% Casein 75% Gelatin
37.5% Casein 62.5% Gelatin
50% Casein 50% Gelatin
62.5% Casein 37.5% Gelatin
75% Casein 25% Gelatin
87.5% Casein 12.5% Gelatin
100% Casein

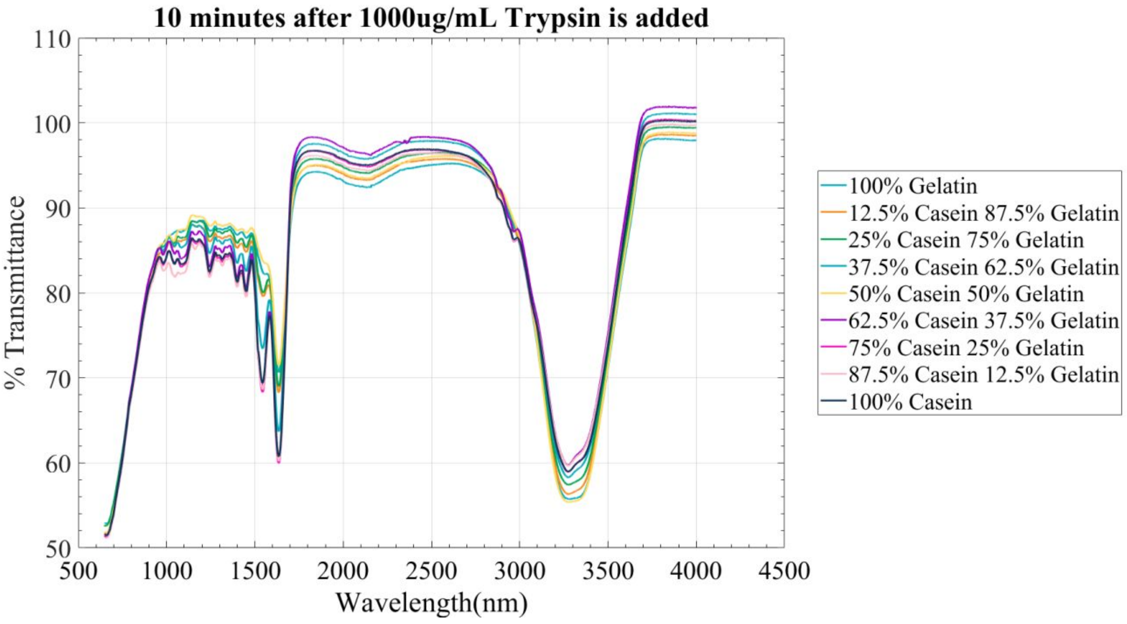

10 minutes after 1000ug/mL Trypsin is added
% Transmittance
Wavelength(nm)
100% Gelatin
12.5% Casein 87.5% Gelatin
25% Casein 75% Gelatin
37.5% Casein 62.5% Gelatin
50% Casein 50% Gelatin
62.5% Casein 37.5% Gelatin
75% Casein 25% Gelatin
87.5% Casein 12.5% Gelatin
100% Casein

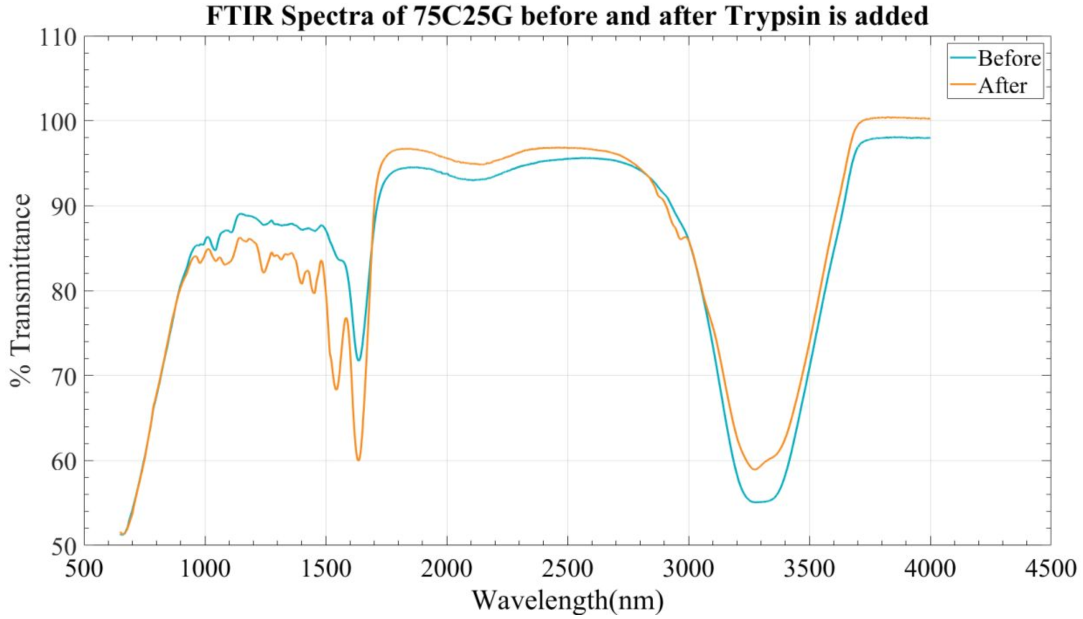

Figure S2. FTIR spectra of blend solutions:(A) Before trypsin addition. (B) 10 minutes after incubation with 1,000 µg/mL trypsin at 37°C. (C) Comparison of the 75C25G optimal blend before and after 10 minutes of trypsin exposure.

(A)

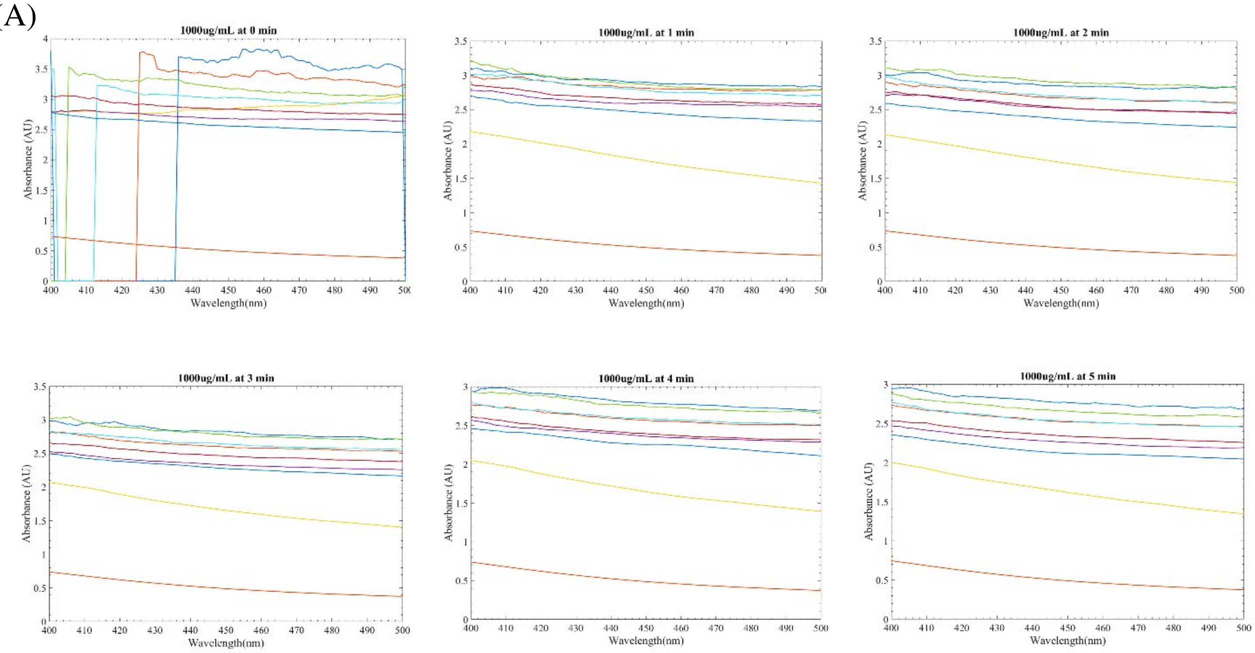

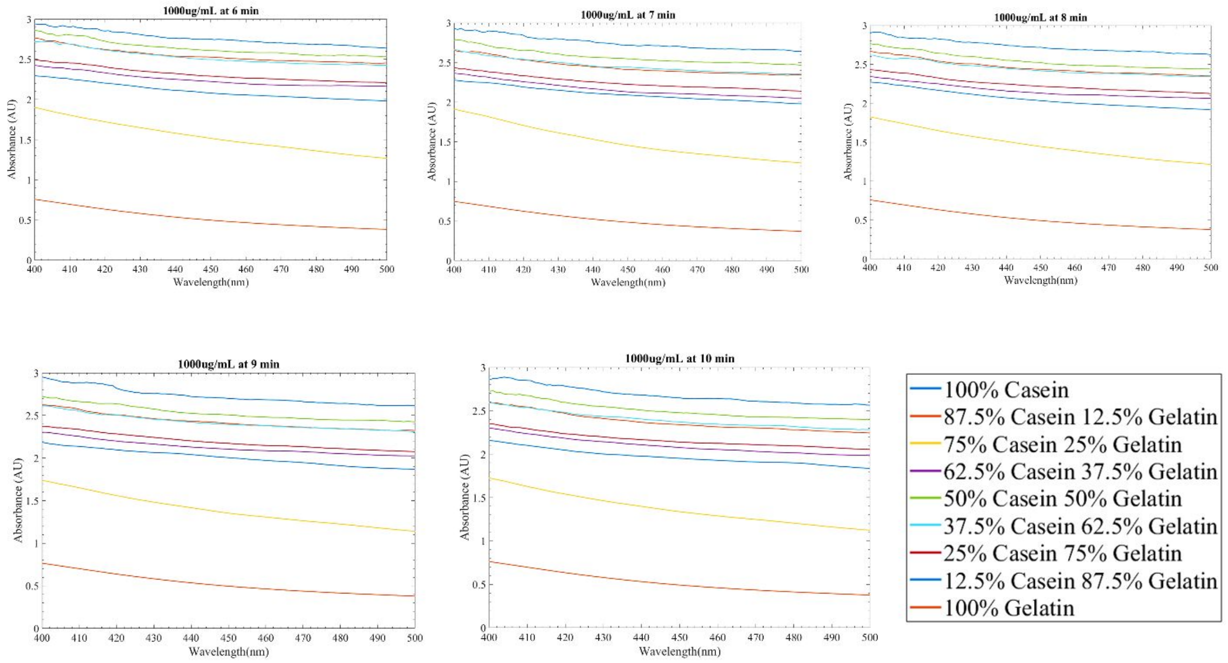

(B)

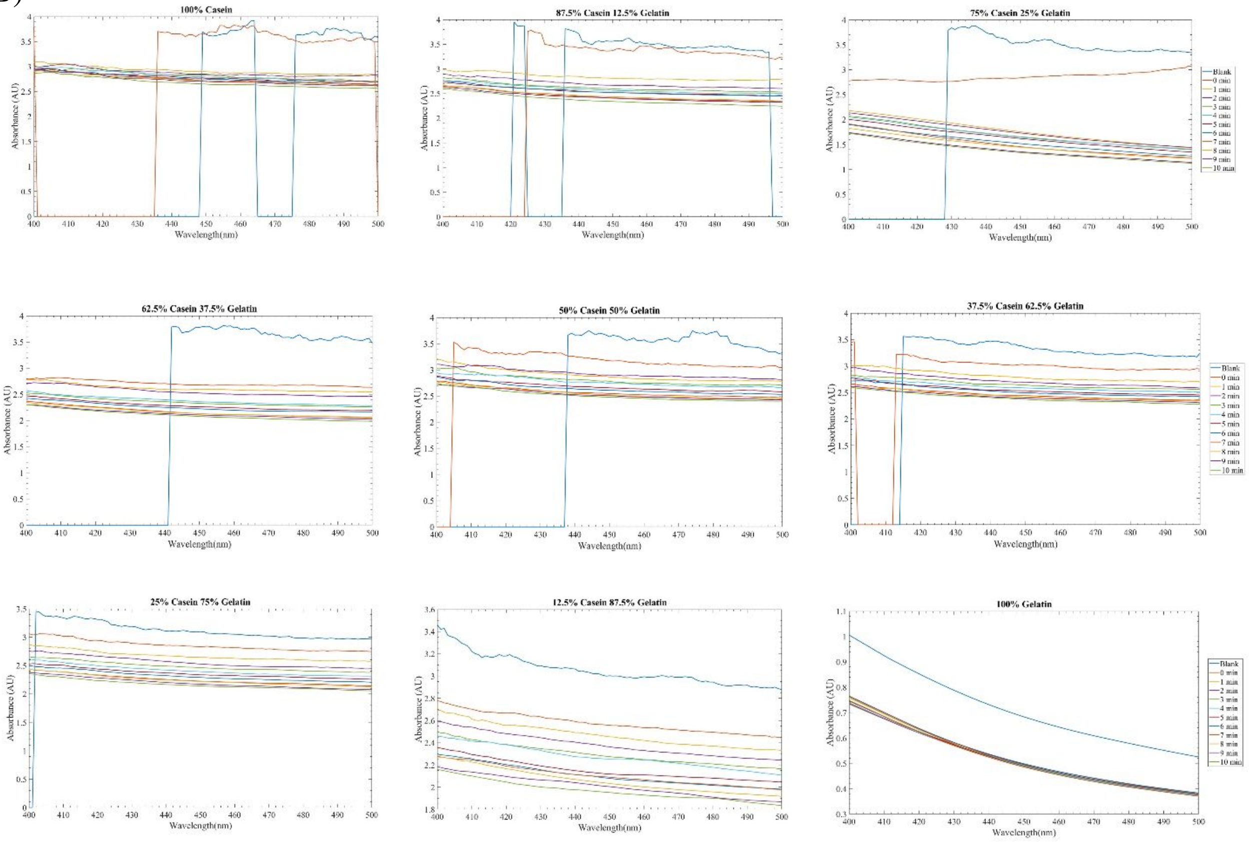

Figure S3. UV-Vis spectrophotometry for Run 1 (400–500 nm): (A) Absorbance spectra of

75C25G from pre-trypsin addition to 10 minutes post-addition, collected at 1-minute intervals. (B) Spectral comparison of all blends over time.

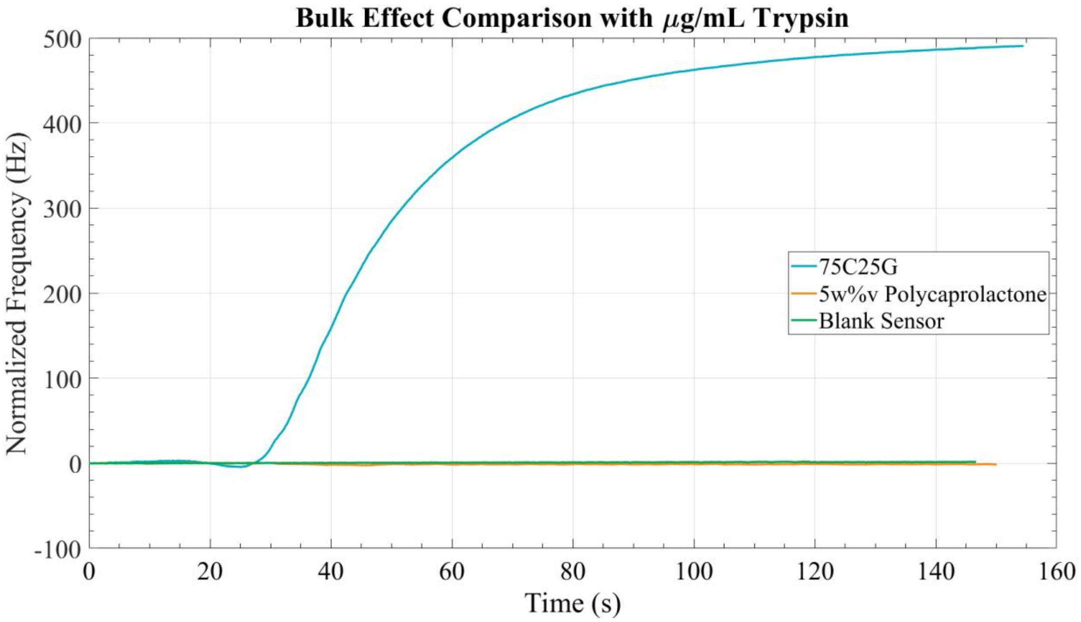

Figure S4. Comparison of frequency responses for the 75C25G blend, 5 w/v% polycaprolactone, and a blank sensor at 1 µg/mL trypsin, normalized to the frequency at pH 5.

Supporting Tables

For each blend and each run (1–3), repeated measures ANOVA was used to test whether there was a **time effect** i.e. whether the absorbance changed significantly over 10 minutes. Table S1 demonstrated the results from the repeated measures ANOVA.

Table S1. Repeated Measures ANOVA for individual blends and runs

		Run 1		***Run 2***		***Run 3***	
Gelatin Content	***Casein Content***	***F-statistic***	***p-Value***	***F-statistic***	***p-Value***	***F-statistic***	***p-Value***
0	*100*	*27370*	*0*	*2332.1*	*0*	*1980*	*0*

12.5	*87.5*	*502.6*	*0*	*203.17*	*0*	*57.01*	*0*
25	*75*	*18566*	*0*	*215.79*	*0*	*1485.7*	*0*
37.5	*62.5*	*133.88*	*0*	*11.919*	*0*	*28.95*	*0*
50	*50*	*353.02*	*0*	*20.721*	*0*	*654.55*	*0*
62.5	*37.5*	*25.367*	*0*	*10.88*	*0*	*12.33*	*0*
75	*25*	*29.487*	*0*	*9.801*	*0*	*32.04*	*0*
87.5	*12.5*	*55.447*	*0*	*60.78*	*0*	*199.2*	*0*
100	*0*	*13.819*	*0*	*5.7878*	*0*	*58.34*	*0*

After which, a grand ANOVA was run to determine if there is a consistent time trend across all three runs.

Table S2. Grand ANOVA across all blends and runs

Gelatin Content	***Casein Content***	***F-statistic***	***p-Value***
0	*100*	*5.096*	*0.0478*
12.5	*87.5*	*2.69*	*0.0202*
25	*75*	*37.49*	*0.028*
37.5	*62.5*	*2.37*	*0.038*
50	*50*	*2.89*	*0.035*
62.5	*37.5*	*2.43*	*0.014*
75	*25*	*2.04*	*0.0009*
87.5	*12.5*	*3.12*	*0*
100	*0*	*0.92*	*0.04*

Table S3. Linear regression analysis of trypsin concentration versus degradation rates.

Gelatin Content	Casein Content	Slope Estimate	RMS Error	F-statistic vs. constant model	p-Value	R-squared
0	100	1.692	0.16	1110.0	5.93E-05	0.997
12.5	87.5	0.523	0.241	47.1	6.33E-03	0.94
25	75	1.419	0.123	1340.0	4.49E-05	0.998
37.5	62.5	0.678	0.292	27.0	3.51E-02	0.931
50	50	0.807	0.477	28.7	1.27E-02	0.905
62.5	37.5	0.285	0.0611	109.0	9.07E-03	0.982
75	25	0.374	0.231	26.4	1.43E-02	0.898
87.5	12.5	0.286	0.0308	173.0	4.83E-02	0.994
100	0	0.023	0.00405	321.0	3.80E-04	0.991

Corresponding Author: Chinaza Ogbonna, chinaza@gatech.edu, (678) 365-1343
